# Designing process and analysis of a new SOI-MESFET structure with enhanced DC and RF characteristics for high-frequency and high-power applications

**DOI:** 10.1371/journal.pone.0301980

**Published:** 2024-04-26

**Authors:** Ahmad Ghiasi, Lewis Nkenyereye, Fawwaz Hazzazi, Muhammad Akmal Chaudhary, Maher Assaad, Abbas Rezaei

**Affiliations:** 1 Department of Electrical Engineering, Kermanshah University of Technology, Kermanshah, Iran; 2 Department of Computer & Information Security, Sejong University, Seoul, Korea; 3 Department of Electrical Engineering, College of Engineering in Al-Kharj, Prince Sattam bin Abdulaziz University, AlKharj, Saudi Arabia; 4 Department of Electrical and Computer Engineering, Ajman University, Ajman, United Arab Emirates; Dayalbagh Educational Institute (Deemed University), INDIA

## Abstract

This research introduces a new designing process and analysis of an innovative Silicon-on-Insulator Metal-Semiconductor Field-Effect (SOI MESFET) structure that demonstrates improved DC and RF characteristics. The design incorporates several modifications to control and reduce the electric field concentration within the channel. These modifications include relocating the transistor channel to sub-regions near the source and drain, adjusting the position of the gate electrode closer to the source, introducing an aluminum layer beneath the channel, and integrating an oxide layer adjacent to the gate. The results show that the AlOx-MESFET configuration exhibits a remarkable increase of 128% in breakdown voltage and 156% in peak power. Furthermore, due to enhanced conductivity and a significant reduction in gate-drain capacitance, there is a notable improvement of 53% in the cut-off frequency and a 28% increase in the maximum oscillation frequency. Additionally, the current gain experiences a boost of 15%. The improved breakdown voltage and peak power make it suitable for applications requiring robust performance under high voltage and power conditions. The increased maximum oscillation frequency and cut-off frequency make it ideal for high-frequency applications where fast signal processing is crucial. Moreover, the enhanced current gain ensures efficient amplification of signals. The introduced SOI MESFET structure with its modifications offers significant improvements in various performance metrics. It provides high oscillation frequency, better breakdown voltage and good cut-off frequency, and current gain compared to the traditional designs. These enhancements make it a highly desirable choice for applications that demand high-frequency and high-power capabilities.

## 1. Introduction

Nowadays, the advancements in nano-electronic technology have a significant impact on the progress of electronic and digital integrated circuits. Improving the dimensions of these components, increasing their operational speed, reducing energy consumption, and enhancing their efficiency enable the production of electronic components with smaller dimensions and greater processing power. Considering the importance of transistors in today’s world as vital components and important priorities in the electronics industry in future decades, the development of transistor technology plays a crucial role in advancing electronic and digital integrated circuits [1]. Transistors, as one of the key components of electronic devices, are highly important in improving their dimensions and efficiency. Among these, transistors with a MESFET structure based on SOI technology are considered to be a promising technology. Silicon-on-Insulator Metal-Semiconductor Field-Effect (SOI MESFET) transistors hold significant importance in modern electronic devices and applications [2–4]. These transistors offer improved performance due to the reduced parasitic capacitance and enhanced isolation provided by the insulating layer. The SOI MESFET technology finds extensive application in high-frequency communication systems, microwave circuits, and power amplifiers. With their superior speed, low power consumption, and high reliability, SOI MESFET transistors play a crucial role in advancing the capabilities of wireless communication, satellite systems, radar systems, and other cutting-edge technologies [5–7].

The SOI-MESFET transistor is composed of a combination of two transistor technologies, the MESFET and SOI, each of which is used separately in the electronics industry [8]. The MESFET represents a subset of the FET family. It possesses distinct areas for the drain and source, with an interceding channel governed by the gate port. The configuration of this channel closely resembles a Schottky junction, characterized by a metal-to-semiconductor interface [8–33]. When a specific voltage is applied to MESFET gate port, the channel’s width and the current carrier values from its source port to its drain port adjust in proportion to the voltage shifts. MESFET transistors offer benefits such as high speed, low power, noise resilience, and longevity. Owing to their sleek configuration, they are perfectly suited for applications in low-noise power and microwave Op-amps and digital electronics [8–33]. The SOI methodology is recognized as a premier choice in the electronics domain because of its remarkable attributes [9]. It features a thin insulating SiO2 layer, commonly referred to as the Embedded Oxide (BOX) layer. This layer isolates the silicon layers from the substrate, positioning the silicon layers atop the BOX layer. SOI technology has notably enhanced silicon chip performance. The chips exhibit high-speed capability owing to minimized leakage current and reduced transistor interference. Other benefits include low noise, latch-up prevention, low power usage, limited leakage current, and resilience to high voltage and temperatures because of robust insulation [9]. Nonetheless, challenges like self-heating and floating-body effects persist [9]. The SOI-MESFET transistor combines the MESFET design with SOI technology. This fusion leverages the best features of both, enhancing the transistor’s precision, build quality, and overall performance. As such, the SOI-MESFET transistor finds utility in a range of applications, from digital electronics and RF power amplifiers to microwave components, and even in fields like medicine and environmental protection [1,8,9]. Like other transistors, however, the SOI-MESFET has its limitations, including a restricted operating voltage, power supply fluctuation sensitivity, self-heating, lower breakdown voltage due to uneven electric field line distribution, and compromised frequency attributes. These problems can be problematic in some applications that require features such as measurement accuracy and high-temperature stability [9–11]. However, despite the improvements in performance and efficiency compared to other transistors, this transistor is still an effective and appropriate selection for many electronic applications. Nevertheless, the use of solutions such as using new materials with suitable electrical properties and introducing new structures can compensate for limitations and problems with parameters such as breakdown voltage, output power, ability to work at different and higher frequencies, improvement of the electric field, etc. In recent years, various studies have been conducted on improving the limitations of MESFET and SOI transistors [10,11]. In this structure, to boost saturation current and enhance breakdown voltage, two insulation regions have been used under both sides of the gate, and an HFP hidden plate has been used in the encapsulated oxide layer (BOX). However, despite these changes, there has been no significant impact on the gain and output power enhancement [12]. To enhance the device’s energy output, this structure incorporates a tiered buried oxide layer under the gate in the channel and replaces a portion of the BOX oxide with n-type silicon doped with impurities. This results in simultaneous enhancement of saturation current and breakdown voltage, leading to an overall improvement of the device’s output power [13]. A transistor with a raised gate and two separate wells made of silicon in buried oxide has been proposed to improve RF parameters. Increasing the channel thickness using an extended gate and a right-side well in the drain has led to an increase in drain current. In this structure, to improve the floating body effect, a well has been used on the left side of the channel, creating an ionization mechanism for capturing holes [14]. Owing to the elevated critical electric field of the silicon oxide layer in this structure, a parallel dual-layer of oxide and nickel metal has been used in the channel to enhance RF performance parameters and breakdown voltage. The nickel used in the channel has helped to increase the scattering of the channel electric potential contours near the gate boundary [15]. To improve the breakdown voltage along with RF characteristics in this structure, different materials are used to generate scattering regarding the electric field trajectories along the channel. This has been achieved by introducing two different trenches in the BOX oxide and transistor channel. The first trench, made of hafnium material, possesses a superior critical electric field. The second trench is made of n-type silicon, identical to the channel’s material, in the oxide area [16]. This paper introduces an innovative technique to enhance the performance of SOI-MESFET devices by repositioning the gate electrode. By altering the placement of the gate electrode compared to the traditional configuration, there’s a marked reduction in the electric field line density within the breakdown voltage area. Such an adjustment translates to an improved conductivity of the transistor, thereby boosting its overall performance [17]. The structure includes a dual-layer to enhance device performance: a nickel metal layer is strategically placed at the channel’s edge within the Buried Oxide (BOX) to optimize electric field distribution, which enhances output power and unilateral power gain. Additionally, the proximity of the silicon oxide layer to the gate and drain terminals serves to increase the device’s resistance to higher electric fields, thereby improving the breakdown voltage as detailed in [18]. Given the limitations of SOI-MESFET transistors and the need for new structures to address these issues, this article proposes a new AlOx-MESFET structure with the goal of increasing the breakdown voltage, current, and output power. Furthermore, this advanced design has contributed to notable advancements in the RF (radio-frequency) specifications. These advancements are crucial for applications that prioritize high-frequency operations. With the superior RF characteristics, the transistor can manage swifter signal transitions, minimizing lags and guaranteeing improved synchronization in both communication and computational endeavors. This not only broadens the scope of applications for these transistors but also underscores their potential in the vanguard of semiconductor innovations, especially within the high-speed communication and sophisticated signal processing sectors.

## 2. The architectural design of the AlOx-MESFET transistor

**Fig 1:**
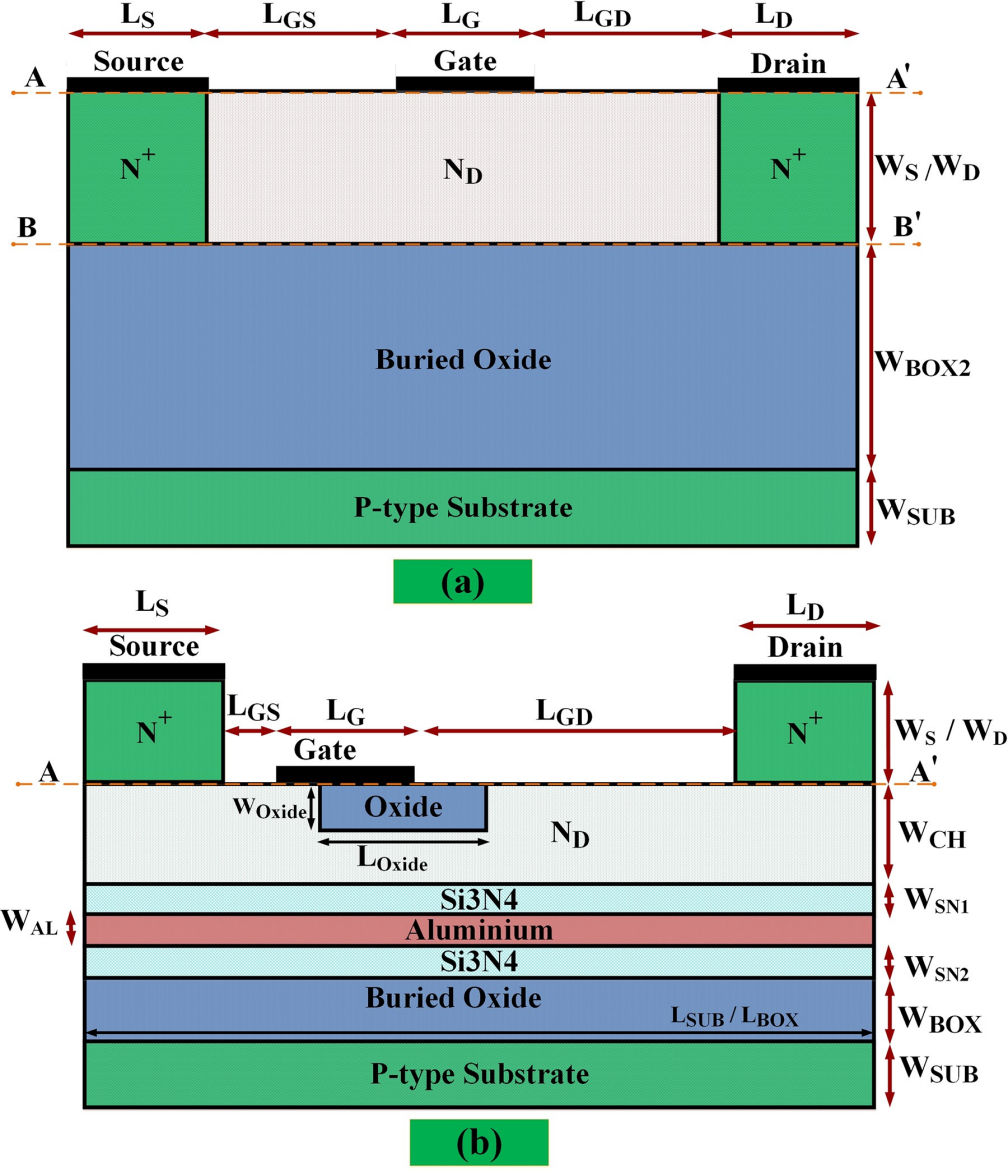
(a) Conventional structure, (b) AlOx-MESFET.

Fig 1, illustrates the conventional SOI-MESFET(C-MESFET) structure in part (a) and the newly proposed AlOx-MESFET structure in part (b). Both structures possess identical vertical dimensions of 0.7 μm and share the same horizontal measurements, amounting to 2.1 μm. The dimensions pertaining to the drain, source, and substrate regions remain consistent across both designs. Nevertheless, the new AlOx-MESFET introduces several modifications when juxtaposed with the traditional structure. These alterations encompass the elimination of the silicon region associated with the channel from the conventional design and the introduction of new regions situated above underneath the source and drain regions, situated within the buried oxide layer. Furthermore, the gate electrode in the updated design is repositioned nearer to the source region. This revised structure also features a reduced buried oxide layer compared to its predecessor, with an added aluminum layer atop it. Concludingly, oxide and aluminum sections are established adjacent to the gate edge and the channel base in the AlOx-MESFET design. In selecting materials for the source, drain, and gate, several key factors such as electrical conductivity, heat resistance, and chemical compatibility with other materials in the device are considered. These materials can include metals like aluminum or semiconductor compounds like doped silicon. The precise choice of these materials contributes to the function and performance of the device. In the presented AlOx-MESFET structure, the compounds used in these areas are similar to those in conventional structures, and the amount of impurities doped into them is provided in Table (1). The use of oxide materials alone cannot positively impact the overall properties of the transistor structure. Therefore, to enhance the overall performance and increase the reliability of the device, other compounds such as aluminum have been utilized in the proposed new structure. The distance of the oxide layer at the edge of the gate is determined based on specific design needs or device optimization. However, the oxide can only be placed at the gate edge to an extent that it does not hinder the gate’s normal functioning and efficiency. The necessity of positioning this oxide layer is due to the higher concentration of electric field lines on that side. The physical principles behind this are optimizing the distribution of the electric field near the gate, preventing current leakage, or enhancing the efficiency of the transistor. The main issue with connecting conventional silicon to aluminum in SOI MESFET transistors relates to the differences in thermal expansion between these two materials. When the device is exposed to varying temperatures, silicon and aluminum expand or contract differently. This disparity in expansion can cause mechanical stress at the junction, ultimately leading to cracking or even separation of the layers, particularly at higher temperatures where thermal stresses are more pronounced.

**Table 1:** Layer dimensions in both the foundational and proposed structures.

Parameters	Symbol	Value
Length of electrodes drain /source/gate	L_S_/L_D_/L_G_	0.3 μm
Width of drain /source	W_S_/W_D_	0.2 μm
Width of SiO2	W_Oxide_	0.05 μm
Length of SiO2	L_Oxide_	0.3 μm
Width of Buried Oxide	W_BOX_	0.11 μm
Width of Buried Oxide 2	W_BOX2_	0.4 μm
Length of Buried Oxide	L_BOX_	2.1 μm
Width of AL	W_AL_	0.05 μm
Width of SN1	W_SNI_	0.04 μm
Width of SN2	W_SN2_	0.02 μm
Length of AL	L_AL_	2.1 μm
Space between gate electrode and drain	L_GD_	1.1 μm
Space between gate electrode and source	L_GS_	0.1 μm
Source/drain doping	N^+^	1×10^20^ cm^-2^
Channel doping (Donor)	N_D_	1.5×10^17^ cm^-2^
Substrate doping (Acceptor)	P	1×10^13^ cm^-2^
Substrate length	L_SUB_	2.1 μm
Width of substrate	W_SUB_	0.1 μm

One solution to this issue is the use of intermediary or transition layers that possess better thermal and mechanical properties and act as a flexible component to compensate for the stress caused by differences in thermal expansion. These layers can be made of materials with thermal expansion coefficients intermediate between silicon and aluminum, providing a smoother transition between these two materials.

Additionally, the use of modern techniques in the manufacturing process, involving low temperatures or specialized bonding processes, can be beneficial. These methods help reduce thermal stresses during thermal processes and provide stronger and more durable connections. Consequently, these approaches can contribute to enhancing the stability and overall efficiency of SOI MESFET transistors.

1. The use of intermediary or transition layers is an effective method to reduce the stress caused by thermal expansion differences between two materials with distinct physical properties, such as silicon and aluminum. These layers act as a buffer and help compensate for thermal variations. The key properties of intermediary layers include:
2. Thermal Expansion Matching: Intermediary layers should have a coefficient of thermal expansion that falls between those of silicon and aluminum. This helps reduce stress at the junction and prevents cracking or separation.
3. Mechanical Flexibility: These layers need to be sufficiently flexible to absorb thermal fluctuations without leading to cracking.
4. Chemical and Electrical Compatibility: In addition to thermal and mechanical properties, intermediary layers must be chemically and electrically compatible with silicon and aluminum to avoid negatively impacting the electrical characteristics of the device.

Materials like Silicon Nitride (Si₃N₄) can serve as suitable intermediary layers due to their good thermal and mechanical properties, acting as a transitional bridge between silicon and aluminum. Choosing Silicon Nitride (Si₃N₄) as the intermediary layer in the proposed AlOx-MESFET structure for SOI MESFET transistors is a wise choice. Silicon Nitride possesses qualities that make it suitable for this application:

1. High Thermal Resistance: It is highly resistant to thermal variations, which helps in reducing the stresses caused by thermal expansion differences between silicon and aluminum.
2. Chemical and Mechanical Compatibility: Silicon Nitride is compatible with both silicon and aluminum, effectively serving as an intermediary without impacting the device’s electrical properties.
3. Suitable Dielectric Properties: Si₃N₄ has dielectric properties that can reduce losses and enhance the efficiency of the transistor.

Utilizing Silicon Nitride as an intermediary layer can improve the overall performance of the SOI MESFET transistor and mitigate issues related to thermal discrepancies between silicon and aluminum. Simulation of semiconductor components is a costly and time-consuming process due to their high complexity and unique physical details. Therefore, numerical simulations using appropriate physical models and powerful software with high accuracy can be a suitable and cost-effective alternative to experimental simulations. These physical models should have high accuracy and the ability to apply physical conditions close to reality. In the described process, several physical models are utilized, such as Bbt.std, SRH, Auger, Fldmob, Analytic and Incomplete, and Impact Selb Conmob. Each model offers its unique method for characterizing and simulating the distinctive attributes of these semiconductor configurations within the Silvaco is ATLAS 2-D simulation tool [19].

## 3. Optimization of the layers in the AlOx-MESFET structure

**Fig 2:**
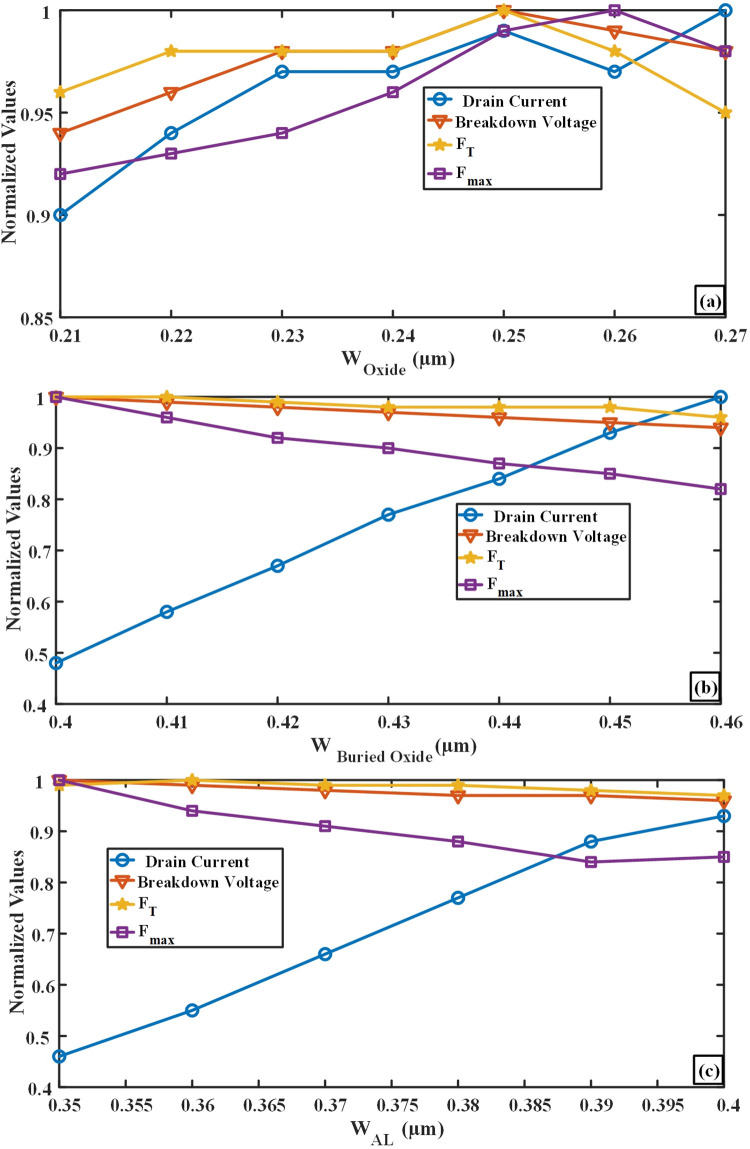
Optimizing of the (a) oxide layer variation range within the gate edge channel, (b) buried Oxide layer variation range, (c) variation range of aluminum layer at the channel’s base.

To obtain optimal and desired outcomes, it’s essential to refine the layers dimensions incorporated into the suggested AlOx-MESFET structure. Hence, this section focuses on optimizing the dimensions of the aluminum, oxide, and Buried Oxide layers in relation to metrics like drain current, cut-off and maximum oscillation frequencies, and breakdown voltage. During this optimization, data was amassed via simulations and subsequently normalized, ensuring a consistent value range. As an outcome of this normalization, the obtained values range between 0 and 1. Fig 2(A) in the proposed transistor demonstrates that the addition of the oxide layer begins at a distance of 0.21 microns with an initial thickness of 0.01 micrometers. This thickness increases to approximately 0.06 micrometers at a distance of 0.27 microns from the device. The considered thickness for this layer in the proposed design is 0.05 micrometers, spanning from 0.2 microns to 0.25 microns. Fig 2(B) shows that the initial thickness of the buried oxide layer, which is 0.2 micrometers, starts at a depth of 0.4 micrometers within the device and continues vertically up to a distance of 0.46 micrometers from the device with a thickness of 0.14 micrometers. For this layer, an optimal thickness of 0.19 micrometers (ranging from a depth of 0.41 microns to 0.6 microns) has been chosen, considering suitable performance and efficiency. Ultimately, Fig 2(C) illustrates the optimization results of the aluminum layer, which is situated along the longitudinal boundary of the channel. The variation in this layer spans from a depth of 0.35 micrometers to 0.4 micrometers in the proposed transistor. The optimal thickness for this layer has been determined to be 0.03 micrometers, extending from a depth of 0.38 microns to 0.41 microns. Table 1 displays the physical dimensions and other impactful parameters of both the proposed design and the conventional structure. Based on the figures provided, variations in the thickness of the oxide, buried oxide, and aluminum layers in the proposed transistor directly affect the drain current, breakdown voltage, cut-off frequency (f_T_), and maximum oscillation frequency (f_max_). The graphs indicate that changes in layer thicknesses influence these parameters differently. For instance, increasing the thickness of the aluminum layer may lead to higher breakdown voltage, f_T_, f_max_, and a decrease in drain current, while changes in the thickness of the buried oxide and oxide layers may have different effects on f_T_ and f_max_.The physical principles behind these changes may include variations in the electric field within the transistor’s channel, the dielectric effects of the oxide layers, and their impact on current distribution and carrier density in the device. Specifically, alterations in layer thickness can change carrier behavior, affecting key parameters like drain current and breakdown voltage. Moreover, precise adjustments to layer thicknesses can optimize the electric field and mitigate undesired field effects, ultimately enhancing f_T_ and f_max_.

## 4. DC analysis of AlOx-MESFET structure

**Fig 3:**
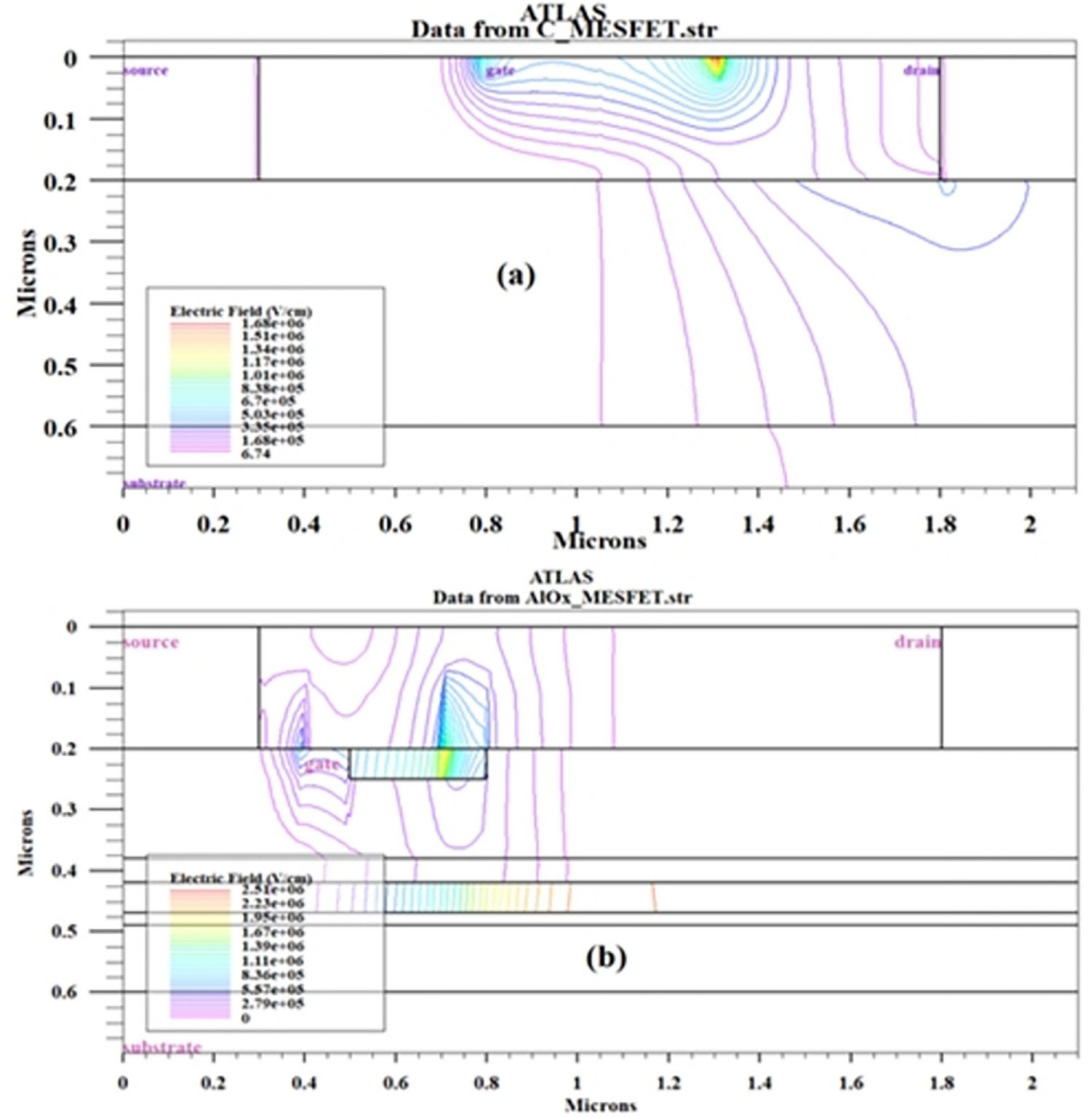
Comparison of electric field line distributions between (a) the conventional MESFET and (b) the presented AlOx-MESFET, at V_GS_ = -3 V and V_DS_ = 12 V.

**Fig 4:**
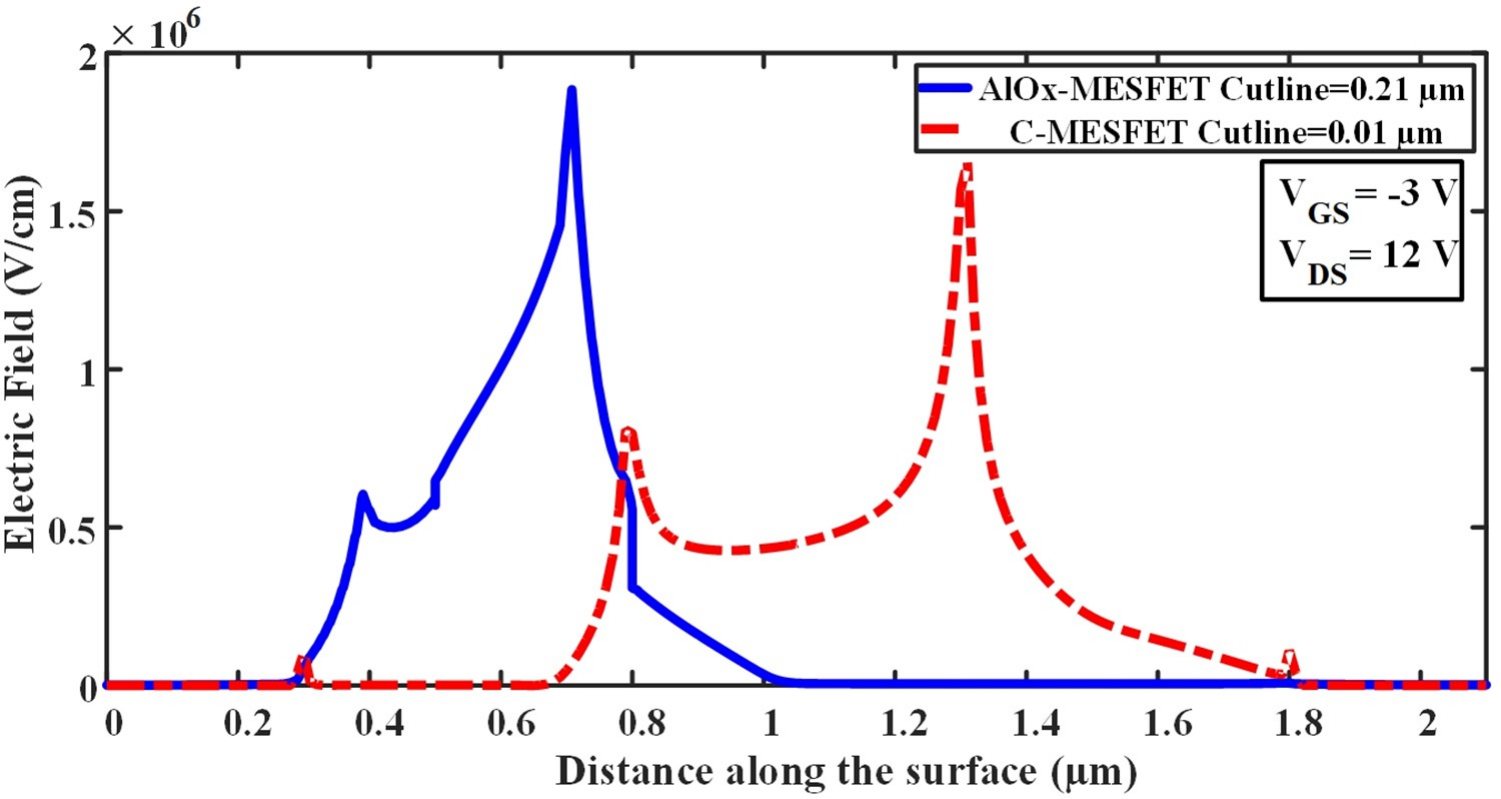
Electric field distribution comparison between the conventional MESFET and the presented structures along the cut line AA’.

**Fig 5:**
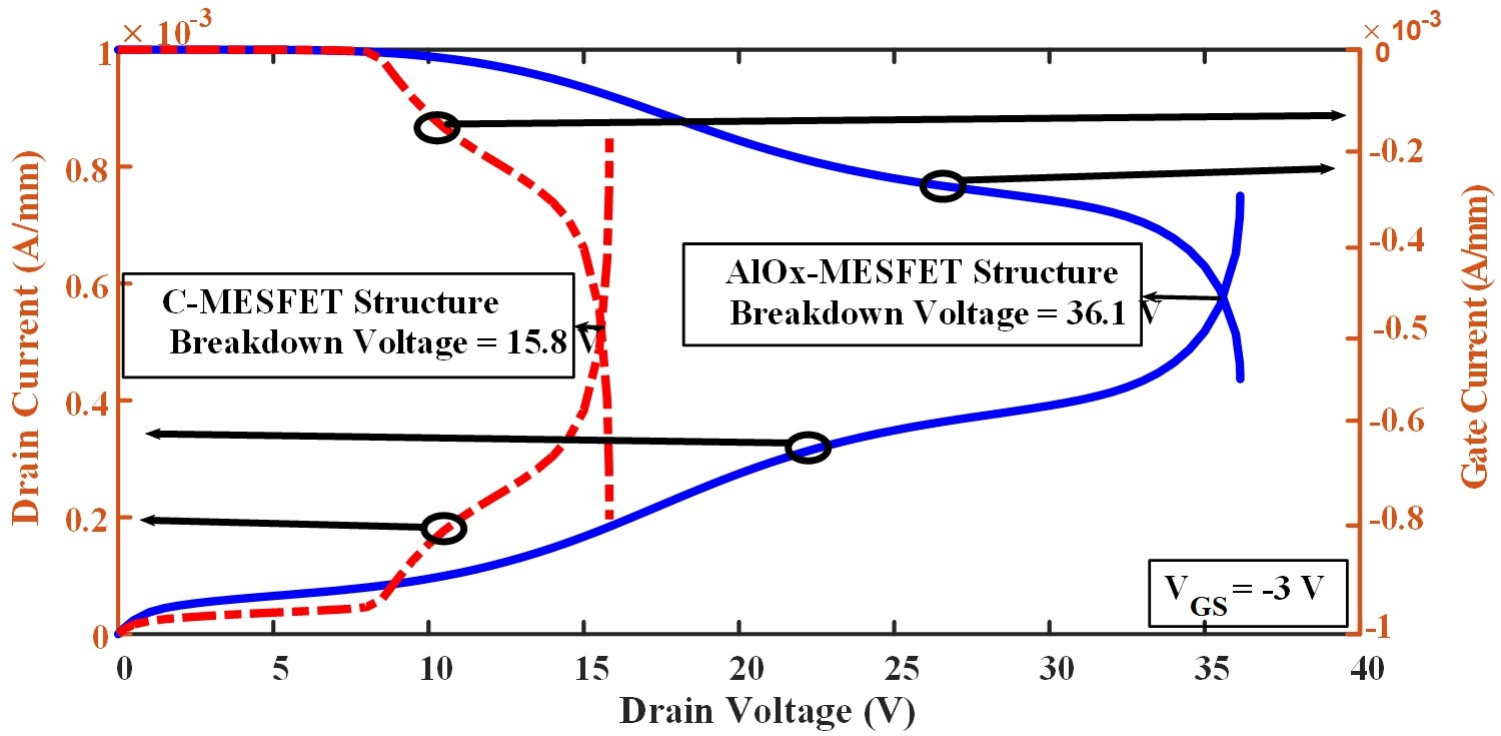
Evaluation of breakdown voltage differences between traditional and AlOx-MESFET configurations.

Voltage breakdown occurs when the applied voltage to a material exceeds its dielectric strength. In Fig 3, part (a) illustrates a standard SOI-MESFET structure where the electric field lines are notably concentrated between the gate region and the boundaries of the drain and source. The amount of charge accumulated in these regions determines the amount of current that can flow through the device. The higher the electric field, the more charge accumulates, and the higher the current that can pass through the device. In a SOI-MESFET transistor, voltage breakdown can occur when the electric field in the device reaches its critical level. This can cause the breakdown of the insulating layer between the gate and the channel, disrupting the structure’s regular pattern and allowing uncontrolled current to pass through the device. To increase the voltage breakdown and maintain proper device operation, the accumulation of electric field lines in these regions should be reduced and precisely controlled [21,22]. To enhance and refine the device design, a novel AlOx-MESFET configuration is introduced. The electric field distribution in the proposed AlOx-MESFET configuration is illustrated in Fig 3(B). To diminish the electric field’s intensity, the transistor channel is repositioned to the under-doped and source regions. This is further augmented by extending the drain to gate distance. This extension is achieved by the shift of the gate port closer to source terminal and incorporating oxide and aluminum layers. Therefore, the electric field’s concentration is lessened, with a portion of it being relocated out of the channel. Furthermore, the incorporation of an aluminum layer at the channel’s base in the suggested structure enhances the absorption and regulation of these electric field lines. This culminates in a reduced electric field concentration and accumulation, leading to a rise in the breakdown voltage and current for this configuration. In order to compare and determine the performance of the electric field in the conventional and proposed structures Fig 4, is presented, which illustrates the electric field at the sub-gate level, following the cut line AA’ as depicted in Fig 1, with V_GS_ = -3V and V_DS_ = 12V. In the conventional structure, the value of this distance is 0.01 micrometers on the surface, while in the proposed structure, it is 0.21 micrometers at the surface distance. In the conventional structure, The electric field concentration spans from the edge of the gate electrode on the source side to the boundary of the drain region. which ranges from 0.65 to 1.8 micrometers, and the peak of this accumulation is between 1.2 and 1.4 micrometers. However, in the suggested design, the electric field concentration is lower than in the conventional structure and is in a smaller range, between 0.3 and 0.8 micrometers. The significant relocation of the electric field peak outside the channel in the proposed device is due to the tailored design of the suggested structure. By adjusting the positioning of layers and the materials utilized, the design improves the distribution of the electric field. This could result in a reduced field concentration within the channel, potentially increasing the device’s stability and lifespan. Fig 5, presents a comparison between the breakdown voltages of the traditional design and the newly proposed models. As seen in these diagrams, the voltage at which the current suddenly increases with a high slope is the breakdown voltage of the structure. This indicates a critical point where the electric field in the conventional SOI-MESFET(C-MESFET) device reaches 15.8 volts and in the proposed AlOx-MESFET device reaches 36.1 volts, causing the insulating layer between the gate and channel to break down and allowing current to flow uncontrollably through the device. However, due to the improved design of the proposed device, the electric field concentration in critical regions has been reduced and more precisely controlled. By comparing these values, the proposed device exhibits a breakdown voltage that is 128% higher than that of the conventional structure, thus improving the performance and reliability of the device.

**Fig 6:**
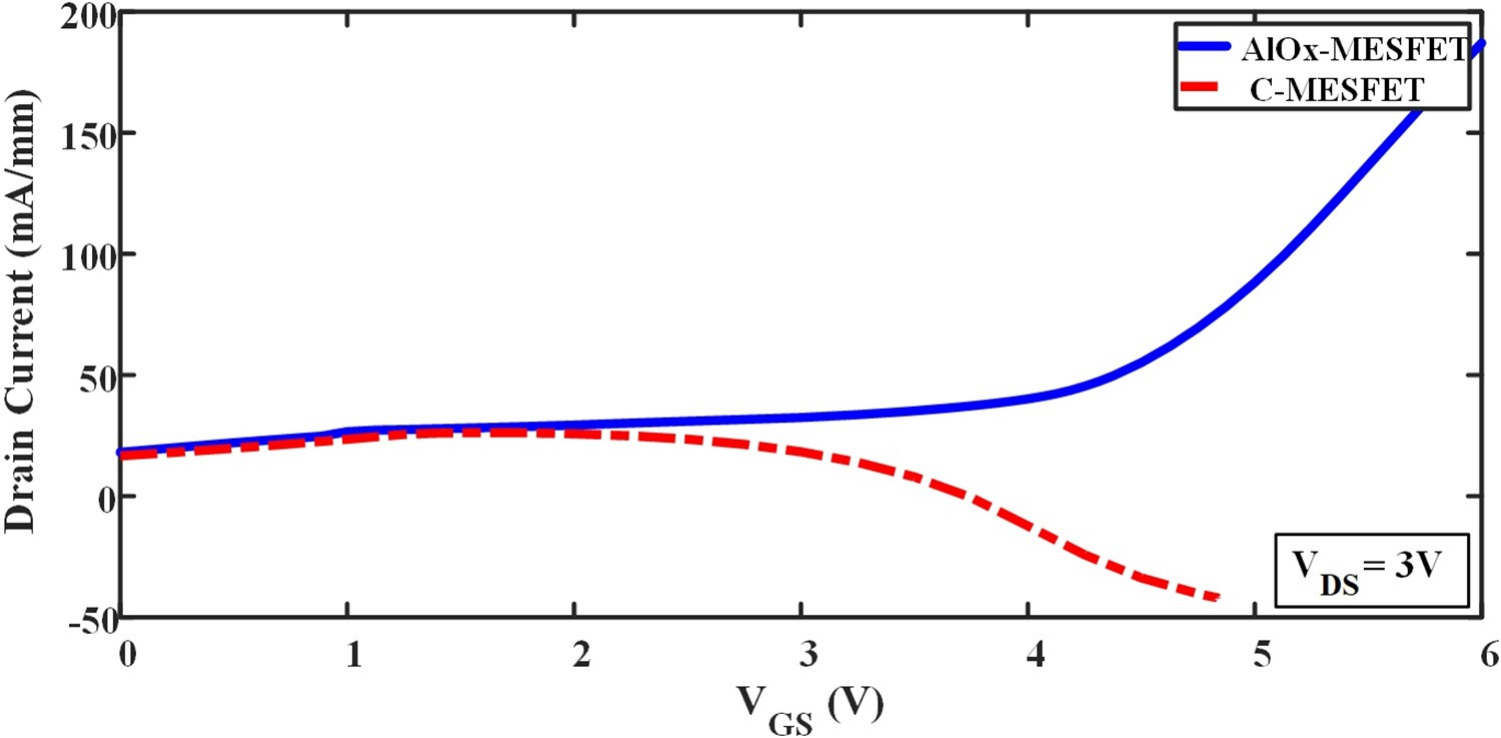
Comparison of gate current of conventional and proposed AlOx-MESFET structure.

Fig 6 the provided Id-Vg graph, we see the drain current versus gate-source voltage curves for both the conventional device (C-MESFET) and the proposed device (AlOx-MESFET). This graph allows for an easy comparison of ION (on-current) and Vt (threshold voltage) values for both devices. It is clear that the proposed device performs better than the conventional one, with higher currents and improved threshold voltages.

**Fig 7:**
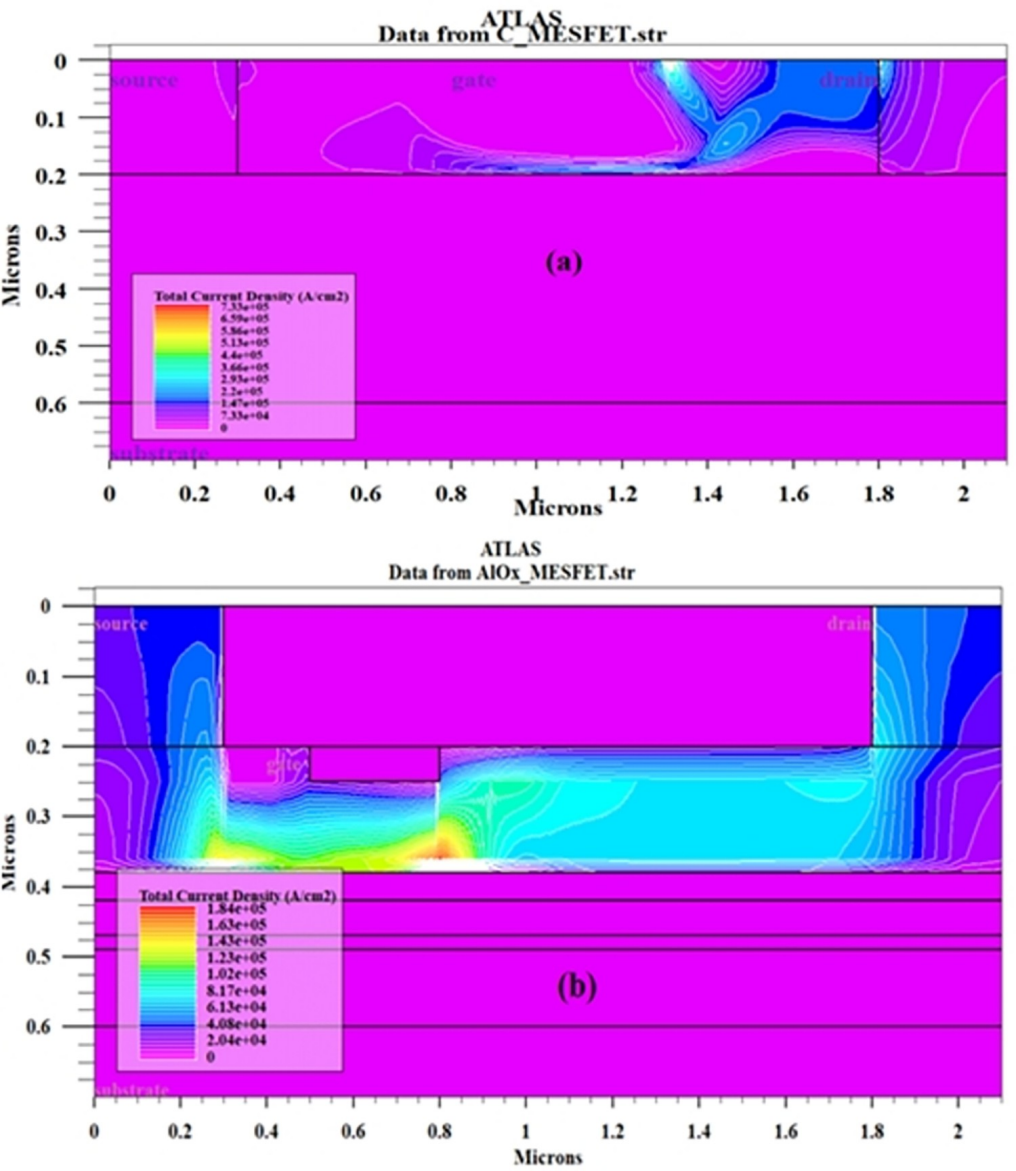
Current density comparison between (a) the conventional MESFET and (b) the presented AlOx-MESFET at V_GS_ = -3V and V_DS_ = 12V.

In the normal state, When the SOI-MESFET transistor structure has no bias voltage applied to its gate, the depletion region remains uniform. However, when a negative bias is depicted in Fig 7, part (a), the depletion region width increases and the amount of electric charge for electrical conduction decreases. This causes a decrease in the effective width of the channel and makes it more compact, which in turn reduces the thickness of the channel and causes carriers to pass through a smaller area, resulting in a decrease in the total current. To further investigate this, Eq (1) [23–34] can be considered:

$$I_{drain}=\frac{Z(h-h(x))\mu n\;e\;Nd\;dv}{dx}$$ (1)

**Fig 8:**
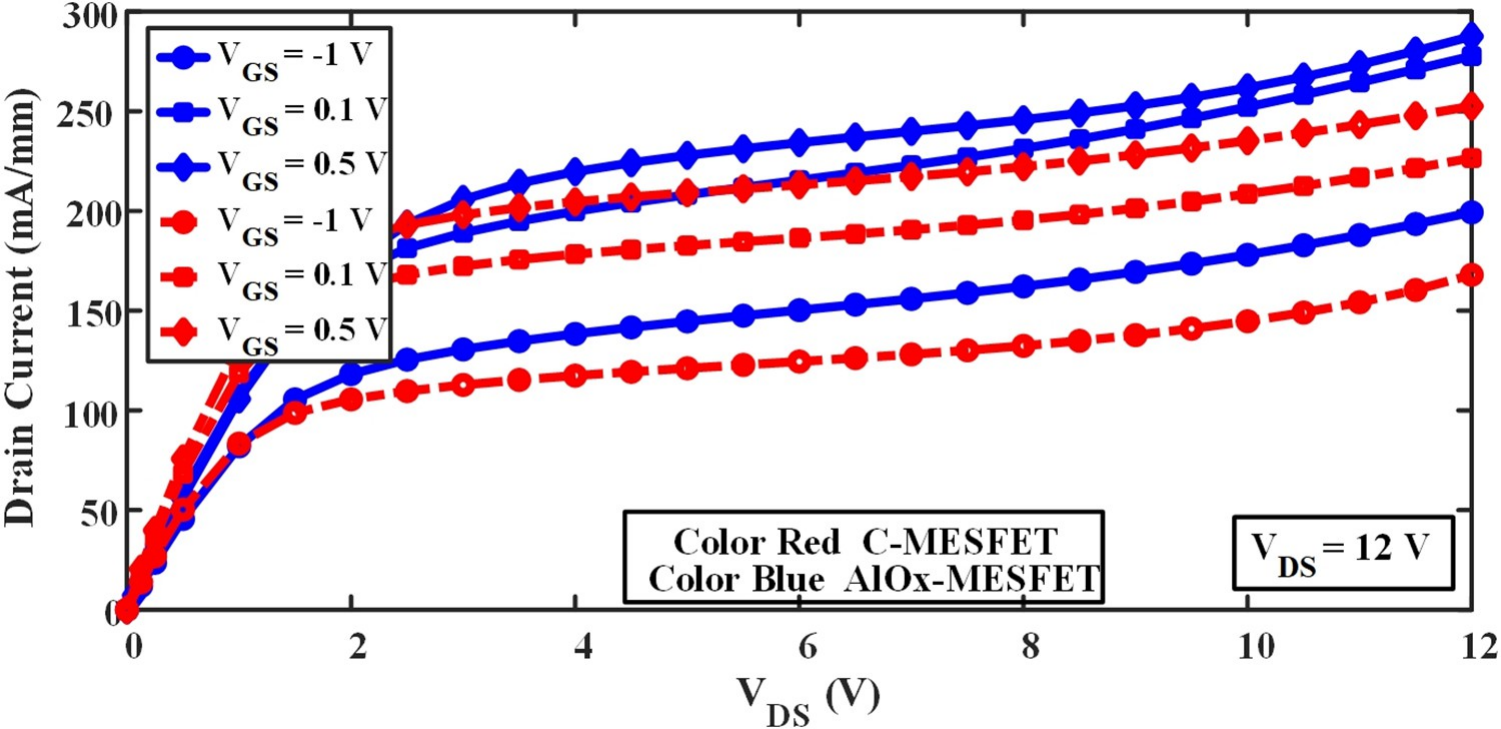
Drain current comparison of the conventional structure versus the AlOx-MESFET structure under different gate-source voltage variations.

In this equation, (e) represents the charge of the electron, (h) denotes the channel thickness, and h(x) signifies the thickness of the depletion section, and Nd represents the concentration value. With an increase in the thickness of the depletion section, the effective width of the channel decreases, which in turn causes a decrease in the current. Based on this, In Fig 7(B), the proposed AlOx-MESFET structure is depicted, by moving the gate port closer to the source terminal and introducing an oxide layer within the channel region, the depletion section width undergoes modification. Consequently, this enhancement in the proposed structures elevates the effective channel width and amplifies the overall current flow. To investigate this issue, Fig 8, has been presented, which shows the I_D_ value versus V_DS_ for the normal structure and AlOx-MESFET structure. The current in this new structure is higher than that in the normal structure with different V_GS_ voltages.

Transistors (SOI-MESFETs) are widely utilized in various electronic applications, notably in certain types of amplifiers. Specifically, when we discuss class (A) amplifiers, a prominent challenge that emerges is their inherently low power efficiency. This inefficiency often limits the performance of electronic devices or systems where they are integrated. One of the critical parameters defining the performance of these amplifiers is the maximum output power. Ensuring a high output power is crucial for the effective functioning of the amplifier in transmitting signals without significant loss. The Eq (2) delineates the method to calculate this maximum output power. By understanding and applying this equation, designers and engineers can devise strategies to enhance the performance and efficiency of the class (A) amplifiers, thereby making the most of the potential of SOI-MESFETs in their applications [25]:

$$P_{max}=\frac{ID\;sat(V_{BR}-V_{Knee})}{8}$$ (2)

Where:

- **I**_**Dsat**_: represents the saturation drain current of the transistor.
- **V**_**BR**_: is the breakdown voltage of the transistor.
- **V**_**Knee**_: denotes the knee voltage, the point where the transistor starts to conduct significantly, leading to a swift rise in its output power.
- The divisor (8) is a constant derived from the characteristics of class (A) amplifiers.

From the equation, it’s evident that enhancing (**P**_**max**_) necessitates increasing both the saturation drain current (**ID**_**sat**_) and **V**_**BR**_ (breakdown voltage), while simultaneously reducing the knee voltage (**V**_**Knee**_). Such optimizations often require structural modifications to the device and the incorporation of alternative materials. In practical terms, the conventional structure has a knee voltage of 1.5 volts. In contrast, the innovative AlOx-MESFET structure boasts a marginally higher value of 1.98 volts. When we analyze the saturation drain current (**ID**_**sat**_) for V_GS_ = -1V and V_DS_ = 12V, the conventional structures exhibit a current of 168 (mA/mm). The proposed structure, however, outperforms this with a current of 182 (mA/mm). Consequently, these differences manifest in the maximum output power: 0.3 W/mm for the conventional structure and a significantly improved 0.77 (W/mm) for the suggested AlOx-MESFET configuration. The AlOx-MESFET structure, with its design improvements, showcases superior performance metrics. This improvement isn’t just nominal but significant, especially when we consider crucial parameters such as knee voltage and saturation drain current. In electronics, any enhancement in power handling capability or efficiency can have profound implications. Higher power output ensures that the device can manage more substantial workloads without compromising performance. This is especially critical for applications where power output directly influences system performance, such as in radio frequency (RF) amplifiers, transceivers, and high-performance computing. Moreover, there’s an additional aspect to consider: the overall robustness of the system. With an increased breakdown voltage, the AlOx-MESFET structure suggests that it can handle higher voltage stresses, making it less susceptible to premature failure or degradation. In the realm of transistors, where miniaturization often challenges performance metrics, the ability to deliver high power in a small form factor can be game-changing. This provides designers with the flexibility to either reduce the size of their systems without sacrificing performance or significantly boost their system’s power in the same footprint. In conclusion, the proposed AlOx-MESFET structure, given its enhanced capabilities and performance metrics, truly stands out as an evolved design. For industries and applications where high power outputs are paramount, this structure offers a promising pathway to achieve those goals.

## 5. Analysis of frequency parameters of AlOx-MESFET structure

In the realm of semiconductor design, SOI-MESFET transistors hold significant promise for applications like high-speed logic circuits and low-noise amplifiers. However, to fully harness their potential, one must deeply understand and optimize their high-frequency characteristics. Key parameters, such as U (unilateral power gain), F_T_ (cut-off frequency), current gain (h21), maximum oscillation frequency (F_max_), and maximum stable gain (GMS) become pivotal in this context. The behavior of these transistors at high frequencies is directly influenced by various factors. Conductivity parameters play an instrumental role, but equally critical are the parasitic capacitances—especially those in the channel, like Cgs (gate-source capacitor) and Cgd (gate-drain capacitor) values. These capacitances can impede the transistor’s high-frequency response, leading to performance degradation in certain applications. For a superior high-frequency performance, designers aim to curtail these parasitic capacitances and amplify the conductivity within the transistors. The relationships governing the maximum oscillation and cut-off frequencies, crucial for design and optimization, are encapsulated in Eqs (3) and (4) [26–36]. A deeper dive into these equations, coupled with rigorous simulations and experimental validations, can pave the way for more efficient and performant SOI-MESFET transistor designs in the future.


$$F_{T}=\frac{gm}{2\pi (Cgs+Cgd)}$$ (3)

$$F_{max}=\frac{F_{T}}{2}\sqrt{\frac{RDS}{RG}}$$ (4)

**Fig 9:**
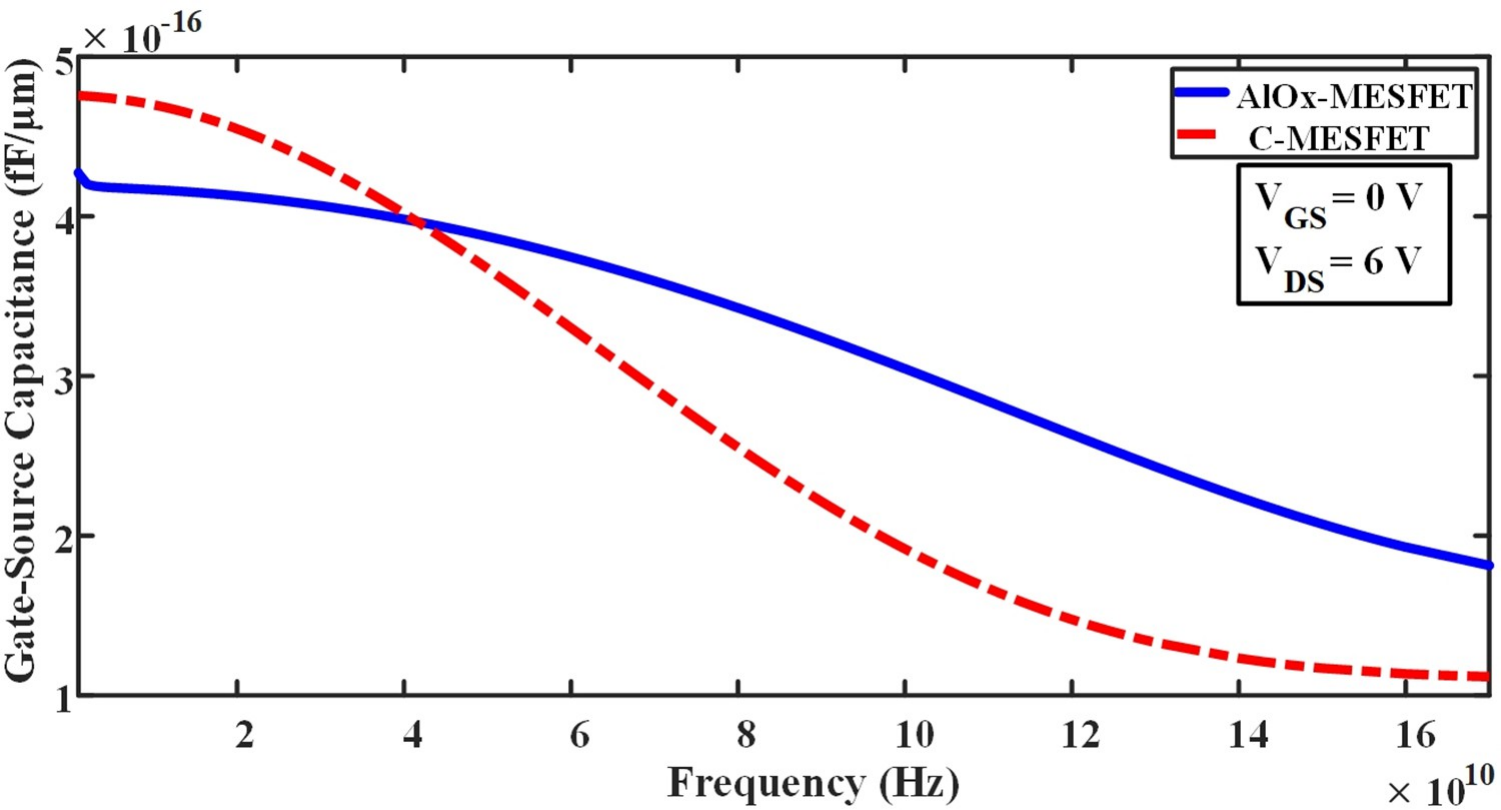
Illustrates the comparison of gate-source capacitance values between the conventional and proposed structures across varying frequencies.

**Fig 10:**
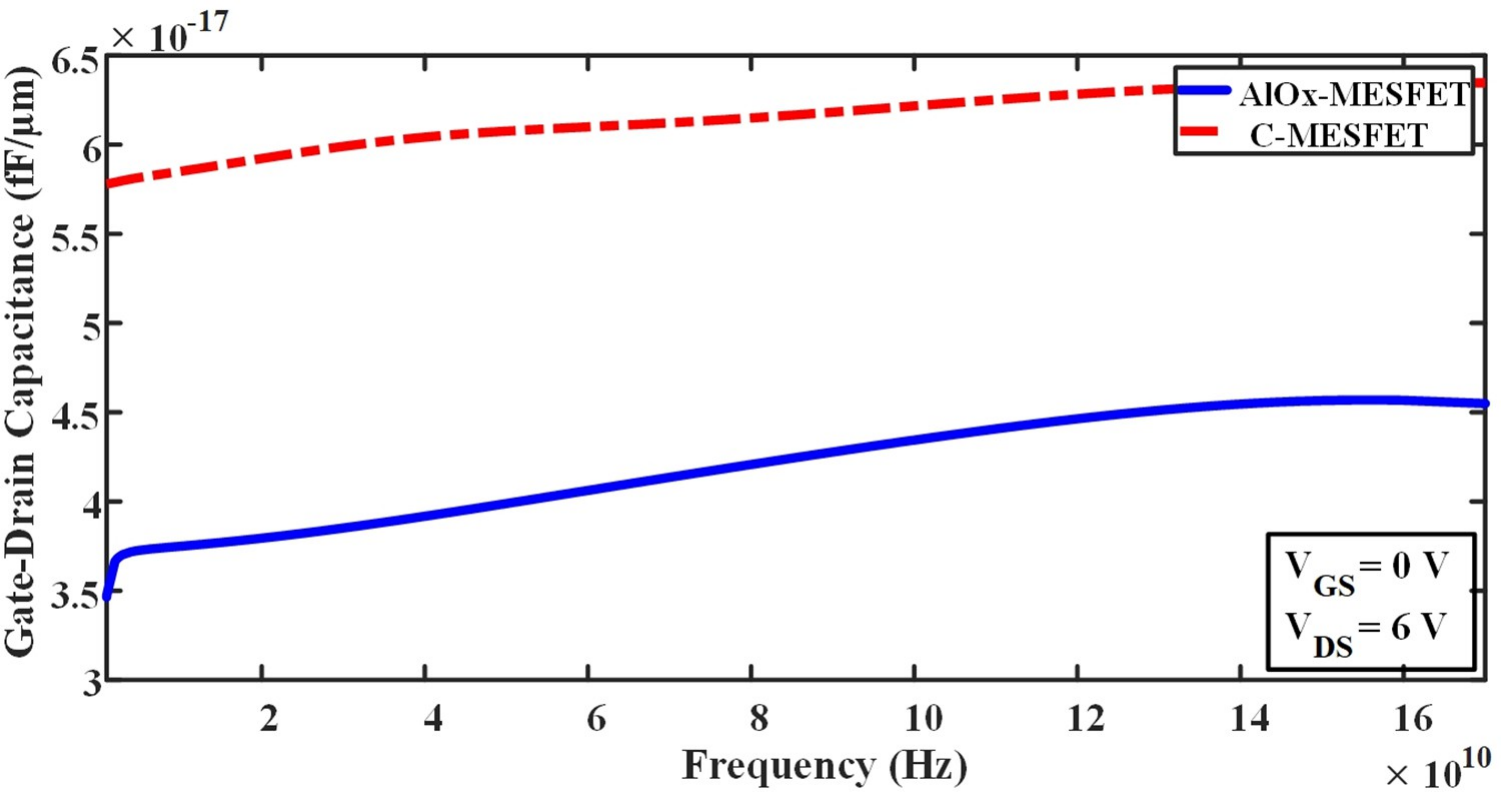
Illustrates the frequency-based comparison of gate-drain capacitance values between the conventional and proposed structures.

**Fig 11:**
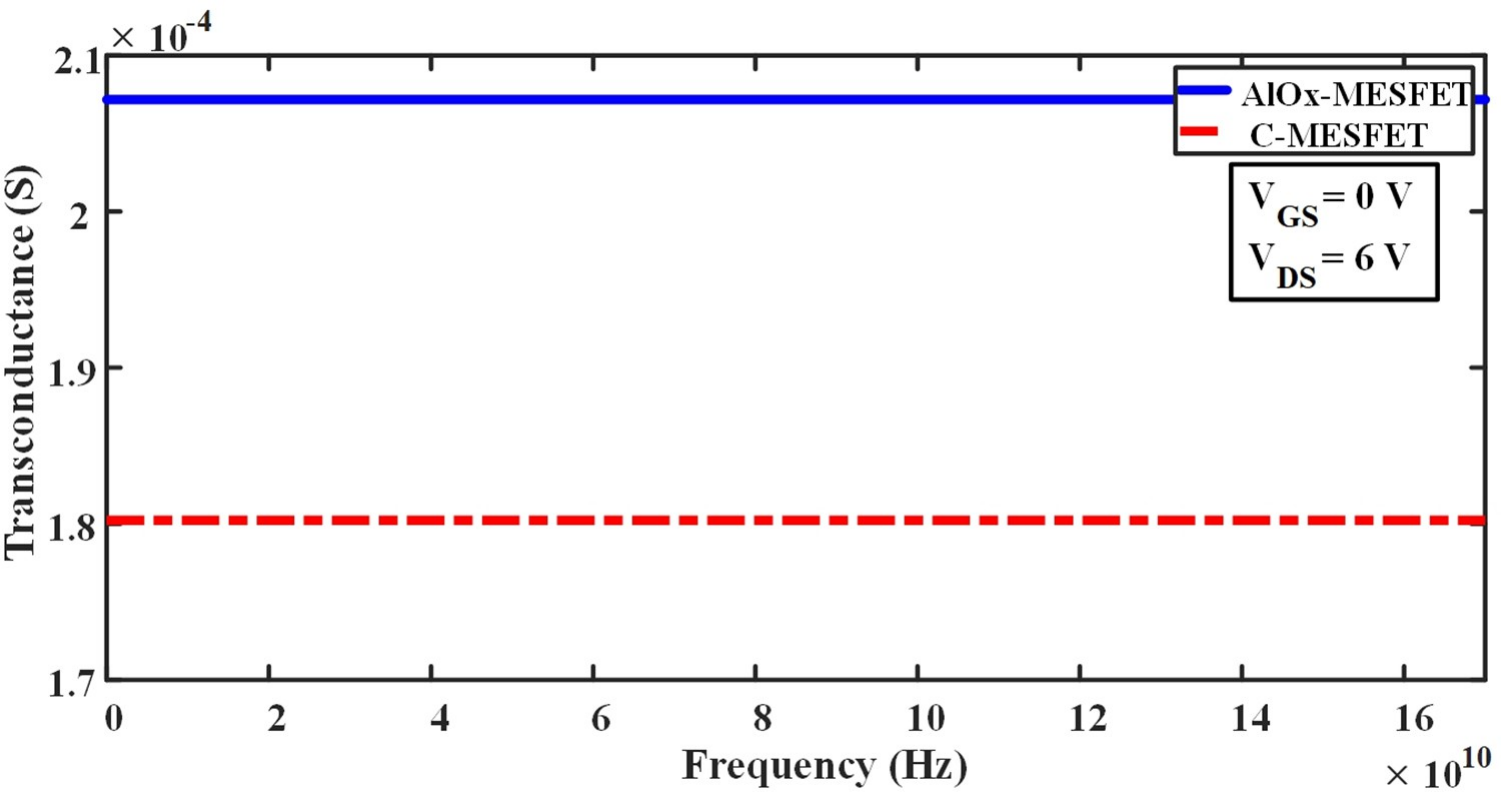
Frequency-based transconductance (S) comparison between the basic and proposed structures.

**Fig 12:**
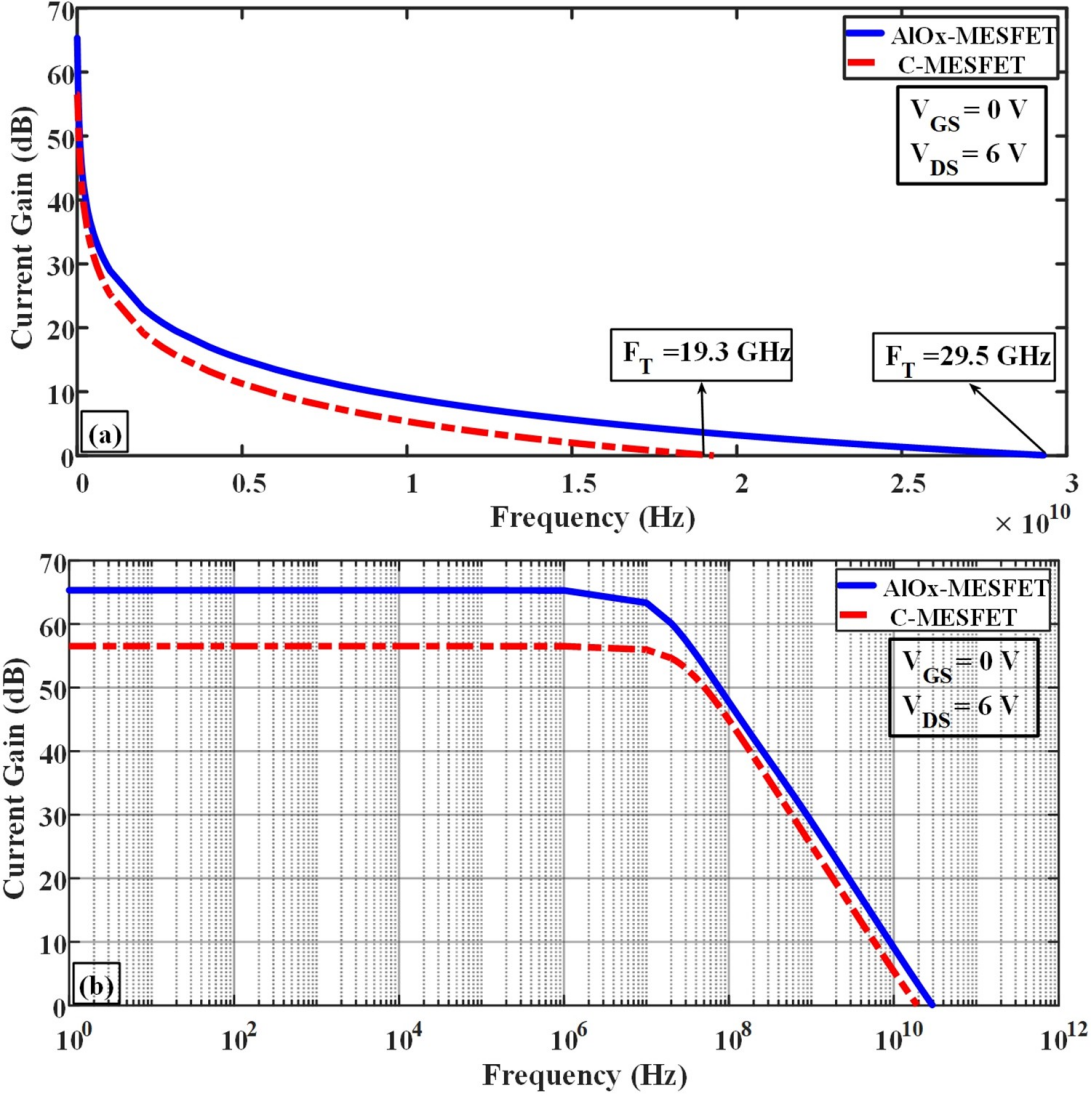
Comparative analysis of current gain between the proposed and traditional structures at various frequencies.

In Eq (4), Where RG represents the resistance of gate, and RDS denotes the resistance of channel, which are obtained from the IG-VG diagram and the ID-VD diagram, respectively [20–35]. By optimizing these parameters and minimizing the parasitic effects, it is possible to improve the frequency response of these transistors and enhance their performance in various applications. The parasitic capacitances play a pivotal role in determining the high-frequency characteristics and performance of SOI-MESFET transistors. In Figs 9 and 10, we contrast the conventional structures against the novel AlOx-MESFET designs in terms of these parasitic capacitances. From the simulations, the proposed AlOx-MESFET structure demonstrates a noticeable reduction in the capacitance of gate-drain (Cgd) when juxtaposed with the conventional model. On the other hand, the capacitance of gate-source (Cgs) remains relatively consistent across both designs. This reduction in Cgd can potentially improve the transistor’s response at high frequencies, making the AlOx-MESFET design a more optimal choice for certain applications. Fig 11, depicts the conductivity (gm) values for both conventional SOI-MESFET(C-MESFET) and AlOx-MESFET structures. From the simulation outcomes, it’s evident that the AlOx-MESFET structure boasts superior conductivity compared to its conventional counterpart. This augmented conductivity suggests potential enhancements in the frequency characteristics of these transistors, making them apt for diverse high-frequency operations. In Fig 12, part (a) shows a comparative plot of cut-off frequency (F_T_) in the conventional and proposed structures. The cutoff frequency values can be obtained directly from the plots without the need for extraction from Eqs (3) and (4). The frequencies indicated correspond to points where the current gain intersects the 0 dB line. In part (b) of Fig (12), a graph displaying a comparison of the logarithmic current gain between the presented structures and the conventional ones is presented. As highlighted in parts (a) and (b) of this figure, the proposed structure demonstrates a notable enhancement in current gain, rising from 56.6 to 65.2 dB, and in F_T_ (cut-off frequency), increasing from 19.3 to 29.5 GHz.

**Fig 13:**
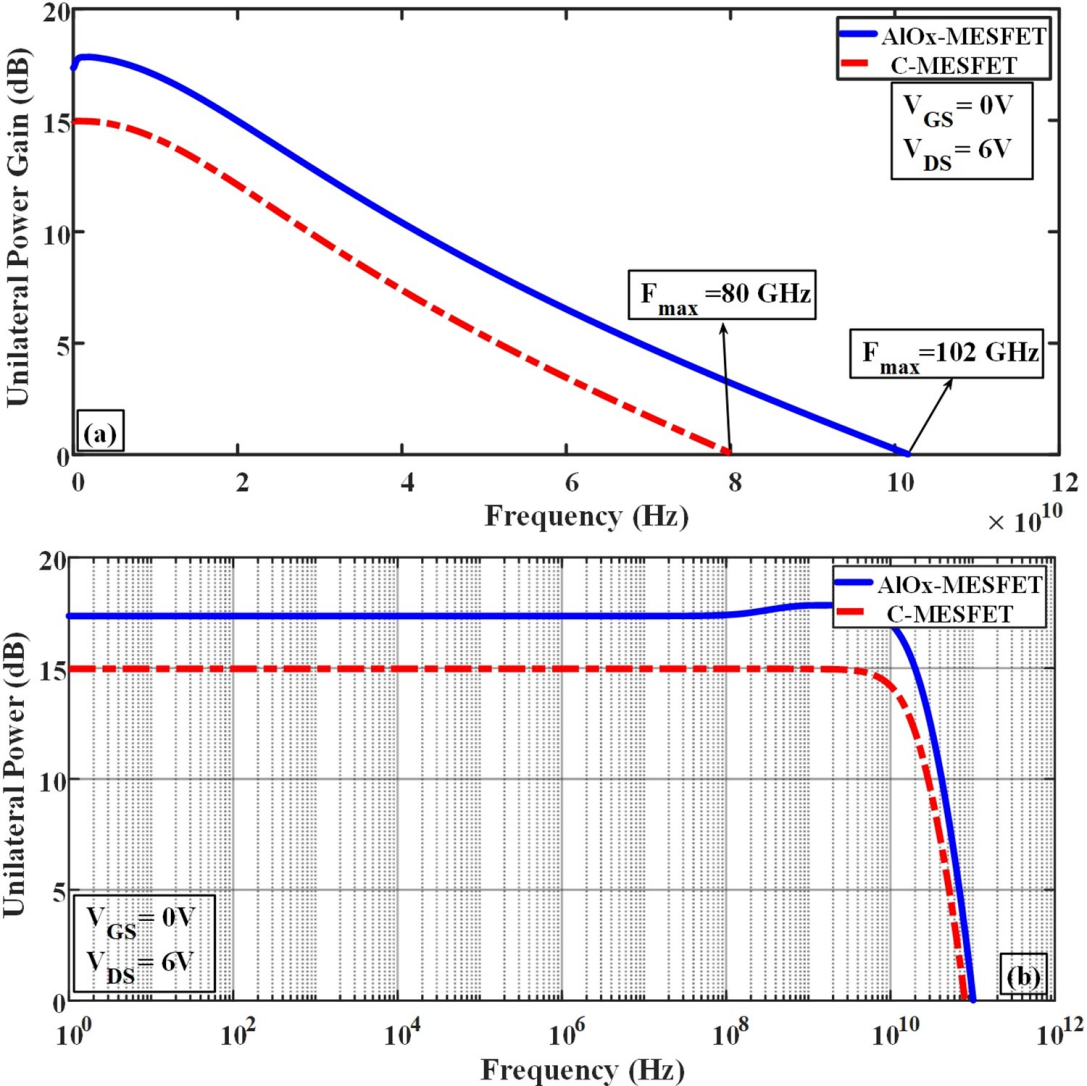
Frequency-based comparison of the unilateral power gain between the presented structure and the conventional ones.

**Fig 14:**
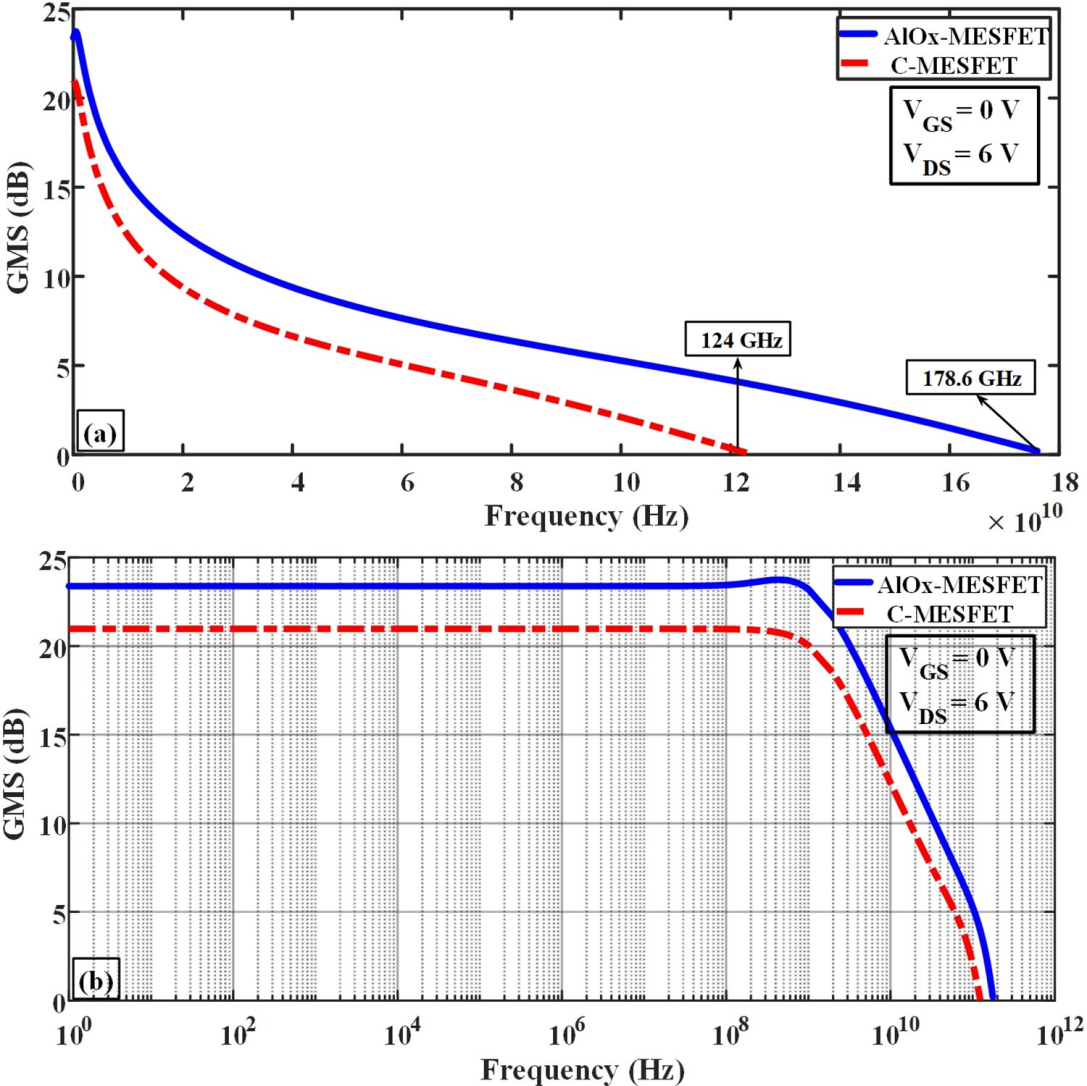
Assessment of the maximum stable gain disparities between the conventional and proposed structures at different frequencies.

In Fig 13, subfigure (a) depicts a comparison of the maximum oscillation frequency (F_max_) between the two structures, identifiable at the point where the graph intersects the 0 dB level. Meanwhile, subfigure (b) of Fig (13) presents a logarithmic representation of the unilateral power gain. The enhanced structure exhibits a noteworthy boost in the unilateral power gain (U), rising from 15 to 17.3 dB, as well as an increase in the cut-off frequency, which shifts from 80 to 102 GHz. Fig 14, parts (a) and (b) show a comparison chart of the maximum stable gain (GMS) in frequency and logarithmic forms, respectively. The proposed structure of AlOx-MESFET has shown a significant improvement compared to the conventional structure. Table 2, provides a general comparison of the presented AlOx-MESFET structure, the conventional configuration and the previous studies (all with the same size). As the results of this table show, the new structure proposed in this article has shown significant improvements compared to previous works, making it a suitable candidate for SOI-MESFET transistors. In terms of breakdown voltage, the proposed AlOx-MESFET achieves a significantly higher value of 36.1 V, compared to the other references which range from 15.8V to 27V. This demonstrates the superior robustness and reliability of our AlOx-MESFET under high voltage conditions. In terms of cutoff frequency, the presented AlOx-MESFET achieves a significantly higher value of 29.5 GHz, compared to the other references which range from 16 GHz to 26.3 GHz. This improvement of 53% enables faster signal processing in high-frequency applications, enhancing the overall performance of our AlOx-MESFET. These advantages make it a highly desirable option for high frequency and power applications, offering improved performance and efficiency in electronic devices and systems.

**Table 2:** Comparison between the proposed AlOx-MESFET structure parameters with those of other structures, as shown in the dimensions of (2.1 μm * 0.7 μm).

Reference	Analysis DC			Analysis AC			
	V_GS_ (V)	V_break_ (V)	P_max_ (%)	V_GS_ (V)	V_DS_ (V)	F_T_ (GHz)	F_max_ (GHz)
Conventional SOI MESFET	-3	15.8	---	0	6	19.3	80
Ref. [12]	-1	23	54.5	0	8	16	96
Ref. [13]	-5	19.6	212	---	---	---	---
Ref. [14]	---	---	143	0	6	20	83
Ref. [17]	-3	27	90	0	8	26.3	117
Ref. [18]	-2	25.8	108	-2	8	19.3	118.5
Ref. [25]	-2	23	109	0	6	24.13	84.9
Ref. [27]	-3	19.5	247	-3	---	25	96
Ref. [29]	-1	22	56	0	8	23	83
Ref. [30]	-3	23	148	0	6	20	91
**AlOx-MESFET in this study**	**-3**	**36.1**	**156**	**0**	**6**	**29.5**	**102**

## 6. Future works

While the proposed structure has demonstrated superior performance in various parameters, there are still areas for further improvement and exploration. One potential area for future work is the optimization of the dimensions of the proposed AlOx-MESFET structure. Further investigation could be conducted to determine if different dimensions could yield even better performance. Another avenue for future research is the investigation of different materials for the oxide layer. While the AlOx layer has shown promising results, alternative materials could potentially offer even higher breakdown voltages or improved RF performance. Furthermore, it would be interesting to investigate the reliability and long-term stability of the proposed AlOx-MESFET structure under high voltage and high-frequency conditions. This could involve accelerated aging tests, thermal cycling experiments, and other reliability studies to assess the robustness and durability of the structure over extended periods of operation.

## 7. Conclusions

Due to the limitations of conventional SOI MESFET transistors such as low breakdown voltage, low maximum output power, and low frequency characteristics, it is essential to improve the parameters of this type of transistors. Therefore, a novel structure is presented to overcome SOI MESFET limitations and improve its performance. In this structure, the breakdown voltage has been improved from 15.8V to 36.1V (an improvement of 128%), saturation current from 168 (mA/mm) to 182 (mA/mm) (an improvement of 8%), maximum output power values from 0.3 to 0.77 (W/mm) (an improvement of 156%), cutoff frequency from 19.3 GHz to 29.5 GHz (an improvement of 53%), maximum oscillation frequency from 80 GHz to 102 GHz (an improvement of 28%), and current gain from 56.6 dB to 65.2 dB (an improvement of 15%). The newly proposed AlOx-MESFET structure with its modifications has successfully overcome the limitations of conventional transistors and demonstrated significant improvements in various performance metrics. The breakthrough enhancements in saturation current, breakdown voltage, cutoff frequency, maximum output power, current gain and maximum oscillation frequency make it an optimal choice for high-power and high-frequency applications. This structure opens up new possibilities for improved performance and efficiency in a wide range of electronic devices and systems.
